# Hemopericardium Following Norovirus Gastroenteritis in a Child: An Uncommon Presentation

**DOI:** 10.7759/cureus.93767

**Published:** 2025-10-03

**Authors:** Maha Hamed, Venkatachalam Karuppaswamy

**Affiliations:** 1 Department of Paediatrics, Tawam Hospital, Al Ain, ARE; 2 Department of Pediatric Cardiology, Tawam Hospital, Al Ain, ARE

**Keywords:** cardiac tamponade, hemopericardium, norovirus, pediatrics, pericardial effusion, viral gastroenteritis

## Abstract

Norovirus is a leading cause of acute gastroenteritis in children and is typically a self-limiting illness. Cardiac complications are exceptionally rare. To our knowledge, this is the first reported case of hemopericardium associated with norovirus infection in a child.

We describe a 20-month-old previously healthy boy who presented with respiratory distress and fatigue two weeks after an episode of acute norovirus gastroenteritis. Investigations revealed severe anemia, elevated D-dimer, and a large hemorrhagic pericardial effusion with echocardiographic evidence of tamponade. Extensive workup excluded trauma, coagulopathy, autoimmune disease, and other infectious causes. The patient underwent urgent pericardiocentesis, draining 200 mL of hemorrhagic fluid. He improved rapidly and remained asymptomatic, with serial echocardiograms showing complete resolution of the effusion up to six weeks post-discharge.

Hemopericardium is a rare but life-threatening complication of pediatric norovirus infection. Clinicians should consider cardiac involvement in children with unexplained cardiopulmonary findings following viral gastroenteritis. Early echocardiography and prompt intervention are essential for favorable outcomes.

## Introduction

Hemopericardium refers to the accumulation of blood within the pericardial sac and may progress to cardiac tamponade, a life-threatening emergency [1]. In children, it is most commonly associated with trauma, cardiac surgery, neoplasms, or anticoagulant therapy [1,2]. Viral causes are extremely rare. Norovirus is among the most common causes of acute viral gastroenteritis in children worldwide, responsible for an estimated 200,000 pediatric hospitalizations annually, and is generally confined to the gastrointestinal tract [3]. Although typically perceived as a mild and self-limiting infection, rare extraintestinal complications such as myocarditis and pericarditis have been reported [4]. To our knowledge, hemopericardium associated with norovirus infection in children has not previously been described.

We present a unique case of hemopericardium following norovirus gastroenteritis in a 20-month-old child, representing the first reported pediatric case and expanding the clinical spectrum of this common viral infection. The patient demonstrated complete clinical recovery with resolution of the effusion on serial echocardiography up to six weeks post-discharge.

## Case presentation

A 20‑month‑old previously healthy boy was brought to the emergency department with respiratory distress, fatigue, and reduced oral intake. Two weeks earlier, he had experienced an episode of acute viral gastroenteritis with vomiting, watery diarrhea, and fever, which resolved spontaneously. There was no history of trauma, bleeding disorders, or anticoagulant use.

On examination, he appeared pale and tachypneic with subcostal retractions. His vital signs were: heart rate (HR) 145/min, blood pressure (BP) 106/75 mmHg, respiratory rate (RR) 47/min, temperature 36.5°C, and peripheral oxygen saturation (SpO₂) 95% on room air (Table 1). Jugular venous distension and muffled heart sounds were noted, while the lungs were clear and the abdominal examination was unremarkable.

**Table 1:** Normal Vital Signs for a 20-Month-Old Child

Parameter	Patient’s Value	Normal Range
Heart rate	145/min	80-130/min
Respiratory rate	47/min	20-30/min
Systolic blood pressure	106 mmHg	85-105 mmHg
Diastolic blood pressure	75 mmHg	35-65 mmHg

The investigations revealed severe microcytic anemia (hemoglobin 6.8 g/dL, decreased from 10 g/dL measured one week earlier at another private hospital) with reticulocytosis and thrombocytosis. The coagulation profile was normal apart from a markedly elevated D-dimer, consistent with active fibrinolysis and recent hemorrhage. Liver, renal, and coagulation studies, G6PD activity, hemoglobin electrophoresis, and blood film were otherwise unremarkable. Carnitine and acylcarnitine profiles were normal, excluding major metabolic defects or fatty acid oxidation disorders. Cardiac biomarkers showed elevated N-terminal pro-B-type natriuretic peptide (NT-proBNP) and troponin-T, indicating cardiac strain with mild myocardial injury. An extensive infectious, metabolic, and autoimmune workup was otherwise negative; however, stool polymerase chain reaction (PCR) performed two weeks earlier was positive for norovirus genogroup II, suggesting a potential viral trigger for the hemorrhagic pericardial effusion (Tables 2-8).

**Table 2:** Key Laboratory Findings Over Time ^*Normal range for a 20-month-old child.^ ^#Absolute reticulocyte count.^ ^†Blood film, G6PD, and hemoglobin electrophoresis were normal at admission (not shown in table).^

Parameter	Admission	Post-Pericardiocentesis	Follow-up (6 wks)	Normal Range*
Red blood cell count (RBC)	2.93 ×10¹²/L	4.56×10¹²/L	4.51×10¹²/L	3.8-5.2×10¹²/L
Hemoglobin	6.8 g/dL	11.5 g/dL	11.1 g/dL	11–13 g/dL
Hematocrit	0.23	0.35	0.35	0.34-0.39
Platelets	643×10⁹/L	620×10⁹/L	237×10⁹/L	150-450×10⁹/L
White blood cell count (WBC)	10.8×10⁹/L	7.4×10⁹/L	8.44×10⁹/L	6-17×10⁹/L
Reticulocyte (%)	6.2%	5.5%	2.0%	0.5-2.0%
Reticulocyte^#^	201.2 ×10⁹/L	180×10⁹/L	60×10⁹/L	25-75×10⁹/L
D-dimer	11,520 ng/mL	8,420 ng/mL	-	<500 ng/mL
Prothrombin time (PT)	11.7 seconds	11.4 seconds	12.1 seconds	10–14 seconds
Internationalized normal ratio (INR)	1.07	1.04	1.11	0.9-1.2
Activated partial thromboplastin time (APTT)	26.3 seconds	24.4 seconds	25.6 seconds	25-35 seconds
Fibrinogen	2.91 g/L	2.91 g/L	2.97 g/L	2-4 g/L
N-terminal pro-B-type natriuretic peptide (NT-proBNP)	1,239 pg/mL	1,108 pg/mL	-	<125 pg/mL
Troponin-T	7.8 ng/L	7.6 ng/L	-	<14 ng/L
C-reactive protein (CRP)	9 mg/L	6.1 mg/L	1.9 mg/L	<5 mg/L
Erythrocyte sedimentation rate (ESR)	81 mm/h	—	18 mm/h	0–10 mm/h

**Table 3:** Electrolytes, Renal Function, and Venous Blood Gas ^*Normal range for a 20-month-old child.^

Parameter	Result	Normal Range*
Sodium	140 mmol/L	135–145 mmol/L
Potassium	5.0 mmol/L	3.5–5.5 mmol/L
Chloride	105 mmol/L	98–107 mmol/L
Carbon dioxide (CO₂)	20 mmol/L	20-28 mmol/L
Creatinine	17 µmol/L	18–35 µmol/L
Urea	1.4 mmol/L	1.8-6.4 mmol/L
Glucose (Random)	5 mmol/L	3.3-5.5 mmol/L
pH	7.43	7.35-7.45
Partial pressure of carbon dioxide (pCO₂)	36.4 mmHg	35-45 mmHg
Partial pressure of oxygen (pO₂)	48.8 mmHg	35-45 mmHg (venous)
Hydrocarbonate (HCO₃⁻)	24 mmol/L	22–26 mmol/L
Base excess	-0.2	-2 to +2 mmol/L
Total hemoglobin (Hb)	7.3 g/dL	11-13 g/dL
Oxygen (O₂) Saturation	83.5%	75-80% (venous)

**Table 4:** Liver Function and Metabolic Workup ^*Normal range for a 20-month-old child.^

Parameter	Result	Normal Range*
Total Protein	35.4 g/L	35–50 g/L
Albumin	28.0 g/L	30–45 g/L
Globulin	7.4 g/L	20–35 g/L
Total Bilirubin	4.5 mg/dL	0.3–1.2 mg/dL
Direct Bilirubin	3.2 mg/dL	0–0.3 mg/dL
Alkaline Phosphatase	229 U/L	150–420 U/L
Aspartate aminotransferase (AST)	23 U/L	10–50 U/L
Alanine aminotransferase (ALT)	22 U/L	5–45 U/L
Carnitine profile	Normal	Normal
Acylcarnitine profile	Normal	Normal

**Table 5:** Other Biochemical Markers ^*Normal range for a 20-month-old child^

Parameter	Result	Normal Range*
Phosphate	1.68 mmol/L	1.3-2.3 mmol/L
Calcium (total)	2.27 mmol/L	2.2-2.7 mmol/L
Magnesium	0.85 mmol/L	0.7-1.0 mmol/L
Lactate dehydrogenase (LDH)	406 U/L	120-300 U/L
Total creatine kinase (CK0	66 U/L	24-170 U/L
Creatine kinase-myocardial band (CK-MB)	<1 U/L	<5 U/L
Triglycerides	0.71 mmol/L	0.3-1.1 mmol/L
Immunoglobulin G (IgG)	7.78 g/L	3.4-12.0 g/L
IgA	0.95 g/L	0.2 – 1.0 g/L
IgM	1.15 g/L	0.3 – 1.5 g/L
Ferritin	66 µg/L	7 – 140 µg/L
Interleukin-6	18.9 pg/mL	< 7 pg/mL
Procalcitonin	0.16 ng/mL	< 0.5 ng/mL

**Table 6:** Pericardial Fluid Analysis

Parameter	Result
Appearance	Bloody
Color	Bloody
Red blood cell (RBC) count	3,320,000/µL
Nucleated cells	5,640/µL
Neutrophils	6%
Lymphocytes	88%
Monocytes	3%
Eosinophils	3%
Albumin	22 g/L
Protein	51 g/L
Glucose	3.0 mmol/L
Lactose dehydrogenase (LDH)	686 U/L
Bacterial and viral polymerase chain reaction (PCR)	Negative
Fluid culture	Negative
Acid-fast bacilli (AFB) smear	Negative
AFB culture	Negative
Cytology	No abnormal cells detected

**Table 7:** Immunology & Serology ANA: Antinuclear antibody; ANCA: antineutrophil cytoplasmic antibody; c-ANCA (PR3): cytoplasmic ANCA (proteinase-3); p-ANCA (MPO): perinuclear ANCA (myeloperoxidase); CMV: cytomegalovirus; EBV: Epstein-Barr virus; Ig: immunoglobulin; Ag/Ab: antigen/antibody; SARS-Cov-2; severe acute respiratory syndrome coronavirus 2; HIV: human immunodeficiency virus.

Parameter	Result
ANA	Normal
c-ANCA (PR3)	Normal
p-ANCA (MPO)	Normal
C3	Normal
C4	Normal
CMV IgG/IgM	Negative
EBV IgG/IgM	Negative
Mycoplasma IgM	Negative
Parvovirus B19 IgM/IgG	Negative
Quantiferon-TB	Negative
HIV Ag/Ab screen	Negative
SARS-CoV-2 total Ab	Negative

**Table 8:** Microbiology and Cultures EBV: Epstein-Barr virus; PCR: polymerase chain reaction; CMV: cytomegalovirus; VRE: vancomycin-resistant *Enterococcus*.

Test	Result
EBV PCR	Not detected
CMV PCR	Not detected
Respiratory PCR	Negative
Blood culture	Negative
Urine culture	Negative
Candida auris screening	Negative
VRE culture	Negative
Stool PCR (2 weeks prior)	Norovirus genogroup II detected

**Figure 1:**
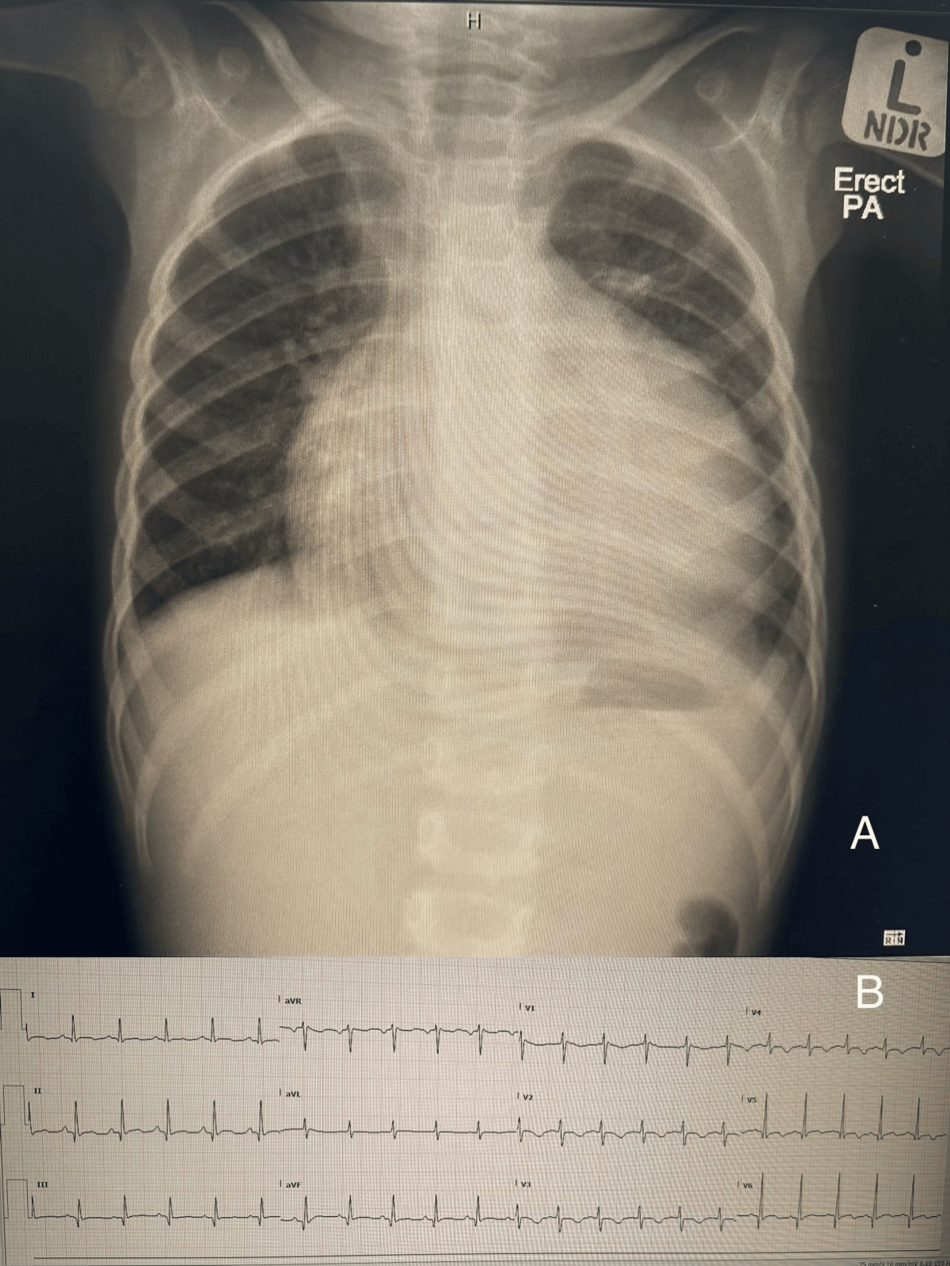
Initial Investigations Panel A: Chest radiograph (posteroanterior (PA) view) demonstrating cardiomegaly with a cardiothoracic ratio of 0.58 (normal ≤0.55 for 20 months of age), consistent with pericardial effusion. Panel B : 12-lead ECG showing sinus tachycardia (~150 bpm) with low QRS voltage in the limb leads, consistent with pericardial effusion.

**Figure 2:**
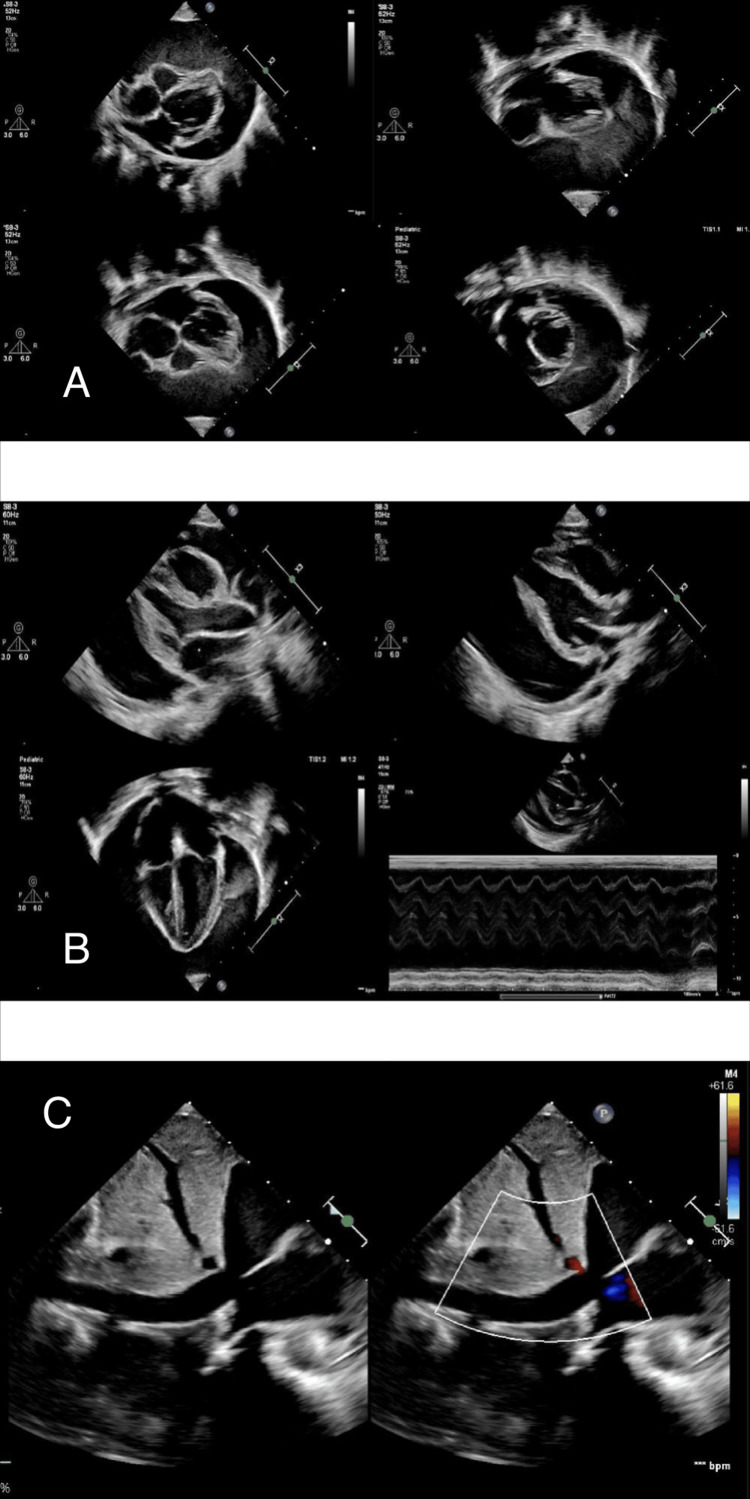
Echocardiography on admission Representative echocardiographic views demonstrating a large circumferential pericardial effusion (2.5 cm) with echogenic densities and tamponade physiology. Panel A: Parasternal short-axis. Panel B: Parasternal long-axis views, apical four-chamber view and M-mode. Panel C : Plethoric (dilated) IVC with reduced inspiratory collapse (<50%), indicating elevated right atrial pressure.

**Figure 3:**
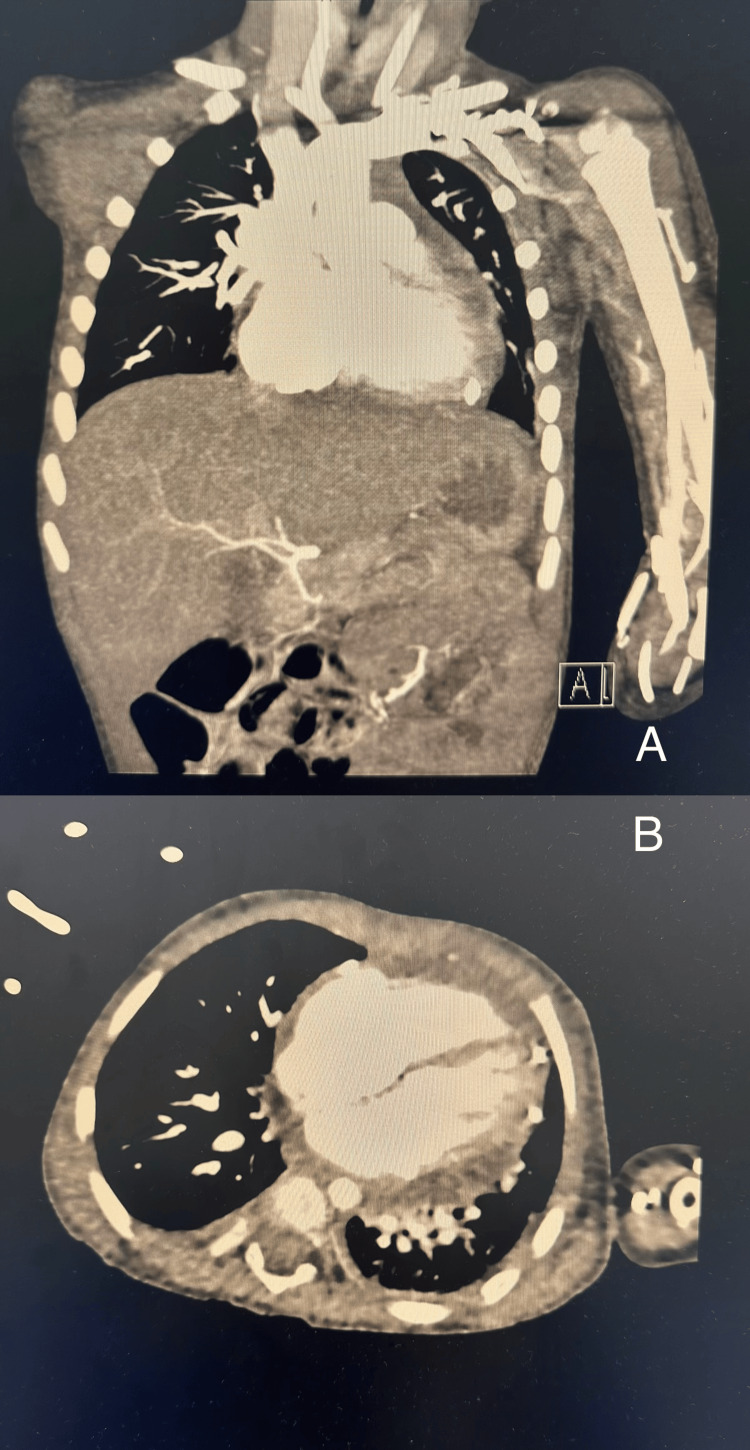
Chest CT Angiography ﻿﻿﻿﻿Panels A and B : Coronal and axial CT views showing large circumferential pericardial effusion without vascular or structural abnormalities. Region of interest (ROI)-based attenuation measurements of pericardial effusion in all views revealed values between 39 and 62 HU, which confirm hemorrhagic fluid. One ROI (179 HU) was excluded as artifact.

Chest radiography demonstrated cardiomegaly, characterized by a globular cardiac silhouette and a cardiothoracic ratio of 0.58 (Figure 1). Electrocardiography revealed sinus tachycardia with low QRS voltage. Transthoracic echocardiography showed a large pericardial effusion measuring approximately 2.5 cm, with echogenic densities suggestive of hemopericardium and echocardiographic features of tamponade physiology (Figure 2). Chest computed tomography (CT) angiography confirmed the presence of a large pericardial effusion without vascular anomalies, with Hounsfield unit (HU) values ranging between 39 and 62, consistent with hemorrhagic content (Figure 3).

The child was admitted to the pediatric intensive care unit (PICU) and managed with supplemental oxygen, intravenous fluids, nonsteroidal anti-inflammatory drugs (NSAIDs), and empirical antibiotics. He subsequently underwent urgent pericardiocentesis, which drained approximately 200 mL of hemorrhagic fluid. Cytological and microbiological analyses of the fluid were negative for bacterial, viral, mycobacterial, and malignant causes. No recurrence of effusion was noted during hospitalization.

**Figure 4:**
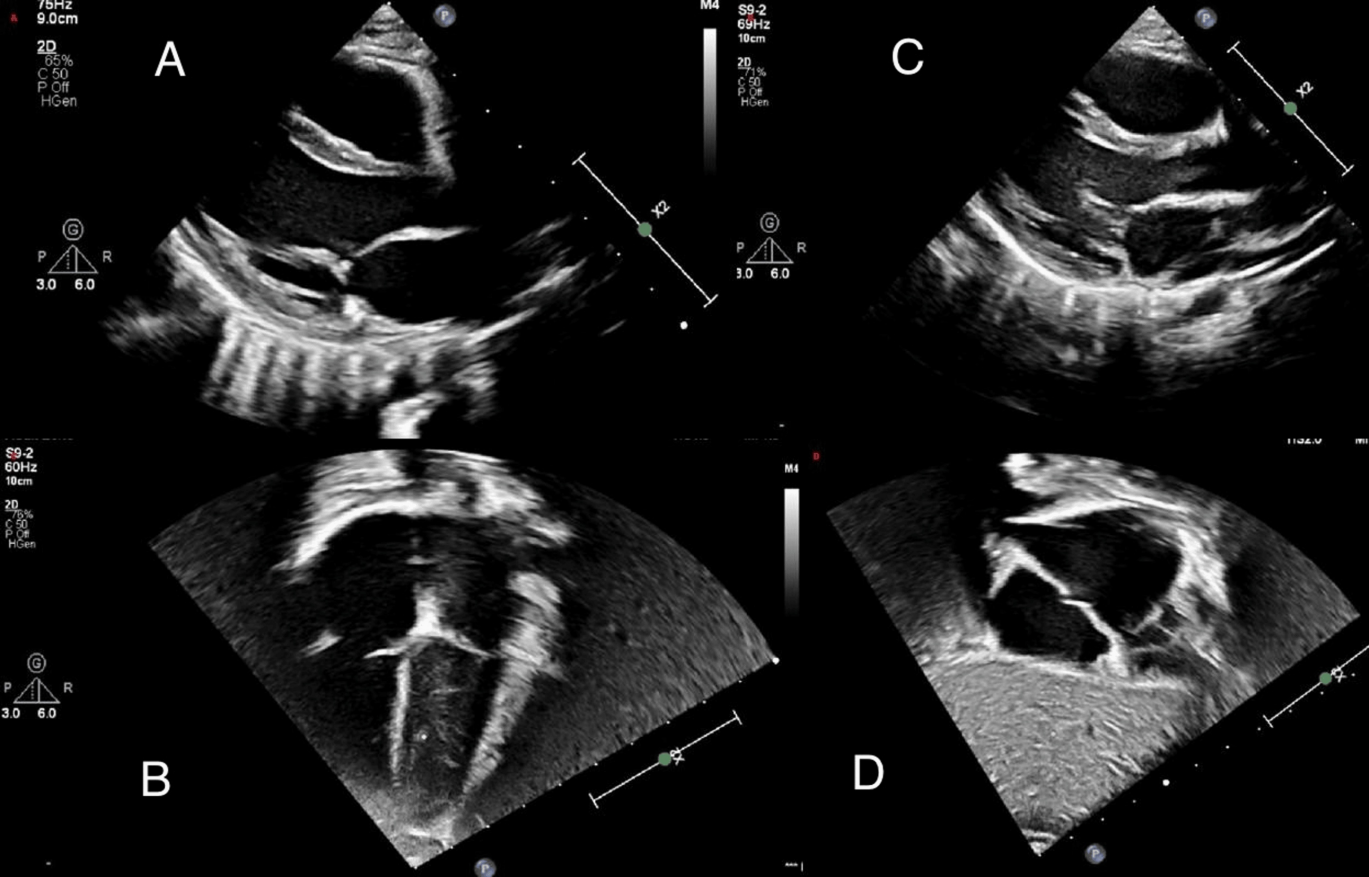
Post-Pericardiocentesis and Follow-Up Echocardiography Panels A, B: Post-pericardiocentesis parasternal long-axis and apical four-chamber views showing resolution of effusion. Panels C, D: Six-week follow-up parasternal long-axis and short-axis views demonstrating absence of residual effusion.

The patient achieved complete clinical recovery and was discharged after 14 days. At six-week follow-up, he remained asymptomatic, and repeat echocardiography demonstrated complete resolution of the pericardial effusion with normal cardiac function (Figure 4). He continues to be followed by pediatric cardiology, with a plan for reassessment and repeat echocardiography at six months post-discharge.

## Discussion

Hemopericardium in children is exceptionally rare and is most commonly associated with trauma, cardiac surgery, anticoagulant therapy, or malignancy [1,2]. Viral etiologies of hemorrhagic pericardial effusion are uncommon but have been reported with pathogens such as coxsackievirus, influenza virus, Epstein-Barr virus, and bacterial gastroenteritis, including *Salmonella* and *Campylobacter* species [4-11]. These cases demonstrate that infections, even outside the cardiovascular system, can occasionally lead to life-threatening cardiac complications.

Norovirus is one of the most common causes of pediatric gastroenteritis worldwide, typically resulting in self-limited gastrointestinal illness [3]. Extraintestinal manifestations are extremely rare. While myocarditis, pericarditis, and cardiogenic shock have been described in pediatric patients following viral gastroenteritis [6], hemopericardium associated with norovirus has not previously been reported. To our knowledge, this is the first documented case of pediatric hemopericardium following norovirus infection, expanding the clinical spectrum of this common viral pathogen.

The pathophysiology of viral-associated hemopericardium remains incompletely understood. Two mechanisms are most plausible: (1) direct viral invasion of the myocardium or pericardium causing local vascular injury and hemorrhagic effusion, and (2) a post-infectious immune-mediated process, consistent with the delayed presentation observed in our patient [7-9]. The markedly elevated D-dimer (11,520 ng/mL) in the absence of systemic coagulopathy suggests localized pericardial endothelial injury with active fibrinolysis. Similar patterns of elevated D-dimer without systemic clotting abnormalities have been reported in viral pericarditis, reflecting localized vascular damage rather than generalized coagulopathy. This finding supports a mechanism of hemorrhagic effusion driven by local inflammation or immune-mediated endothelial injury. Similar immune-mediated mechanisms have been described in bacterial gastroenteritis complicated by myocarditis or pericarditis [5,11-13].

While norovirus was detected in stool two weeks prior, viral RNA was not identified in pericardial fluid or blood, limiting definitive attribution. Rigorous exclusion of alternative causes - including trauma, cardiac surgery, anticoagulant therapy, malignancy, and other viral or bacterial infections - supports a possible association rather than a confirmed causality. The temporal sequence of gastroenteritis followed by hemopericardium, combined with consistency with known viral pericarditis mechanisms, strengthens the inference, though it remains at a “possible” level. Future investigations, such as pericardial fluid PCR or metagenomic sequencing, or paired serology, could provide stronger evidence for causal linkage.

Although other viral infections, such as COVID-19, have occasionally been associated with pediatric pericardial effusions, the mechanisms of viral-induced hemopericardium may be similar across different pathogens. Clinically, pediatric hemopericardium often presents with nonspecific symptoms, including fatigue, respiratory distress, pallor, and tachycardia, which can mimic myocarditis, purulent pericarditis, or malignancy. This underscores the importance of echocardiography as the primary diagnostic tool to evaluate effusion size, cardiac tamponade, and ventricular function [1,2]. In our patient, rapid echocardiographic diagnosis enabled timely pericardiocentesis and complete recovery [4].

Management requires individualized assessment based on hemodynamic stability, with urgent pericardiocentesis indicated in tamponade, along with supportive care and monitoring of inflammatory markers [1,2]. Our case highlights that viral-associated effusions may resolve completely with prompt intervention [12,14-16].

Long-term cardiology follow-up is important, as delayed complications may occur even when the acute course resolves favorably. Further research is needed to clarify the mechanisms by which norovirus may cause cardiac involvement and to guide optimal follow-up strategies in affected children.

## Conclusions

Although norovirus is typically a self-limited gastrointestinal pathogen in children, this case demonstrates a rare hemopericardium possibly associated with norovirus infection. Pediatricians and emergency physicians should maintain a high index of suspicion for cardiac involvement in children presenting with unexplained cardiopulmonary signs following viral gastroenteritis.

Early recognition with echocardiography and timely pericardiocentesis are critical for diagnosis and survival. Awareness of such rare complications allows for rapid intervention, preventing life-threatening outcomes. To our knowledge, this is the first reported case of hemopericardium associated with norovirus. Additional case reports and studies are needed to better understand the underlying pathophysiology, long-term outcomes, and optimal follow-up strategies for viral-associated hemopericardium.
